# Spica Prunellae Extract Enhances Fluorouracil Sensitivity of 5-Fluorouracil-Resistant Human Colon Carcinoma HCT-8/5-FU Cells via TOP2*α* and miR-494

**DOI:** 10.1155/2019/5953619

**Published:** 2019-09-30

**Authors:** Yi Fang, Chi Yang, Ling Zhang, Lihui Wei, Jiumao Lin, Jinyan Zhao, Jun Peng

**Affiliations:** ^1^Academy of Integrative Medicine, Fujian University of Traditional Chinese Medicine, Fuzhou 350122, China; ^2^Fujian Key Laboratory of Integrative Medicine on Geriatrics, Fujian University of Traditional Chinese Medicine, Fuzhou 350122, China; ^3^Institute of Edible Fungi, Fujian Academy of Agricultural Sciences, Fuzhou 350003, China

## Abstract

The use of 5-fluorouracil (5-FU) has been proven benefits, but it also has adverse events in colorectal cancer (CRC) chemotherapy. In this study, we explored the mechanism of 5-FU resistance by bioinformatics analysis of the NCBI public dataset series GSE81005. Fifteen hub genes were screened out of 582 different expressed genes. Modules of the hub genes in protein-protein interaction networks gathered to TOP2*α* showed a decrease in HCT-8 cells but an increase in 5-FU-resistant HCT-8/5-FU cells with 5-FU exposure. Downregulation of TOP2*α* with siRNA or miR-494 transfection resulted in an increase of cytotoxicity and decrease of cell colonies to 5-FU for HCT-8/5-FU cells. Moreover, we found that an ethanol extract of Spica Prunellae (EESP), which is a traditional Chinese medicine with clinically beneficial effects in various cancers, was able to enhance the sensitivity of 5-FU in HCT-8/5-FU cells and partly reverse the 5-FU resistance effect. It significantly helped suppress cell growth and induced cell apoptosis in HCT-8/5-FU cells with the expression of TOP2*α* being significantly suppressed, which increased by 5-FU. Consistently, miR-494, which reportedly regulates TOP2*α*, exhibited reverse trends in EESP/5-FU combination treatment. These results suggested that Spica Prunellae may be beneficial in the treatment of 5-FU-resistant CRC patients.

## 1. Introduction

Colorectal cancer (CRC, also known as colon cancer) is one of the most commonly diagnosed cancers. The GLOBOCAN 2018 data estimates of cancer incidence and mortality showed that CRC is the third most commonly diagnosed cancer, with an incidence of 10.2% and a mortality of 9.2% [1]. Presently, the treatment for CRC includes surgery, radiation, and chemotherapy palliative care [2]. Chemotherapy drugs for the treatment of CRC include capecitabine, oxaliplatin, fluorouracil (5-FU), leucovorin (folinic acid), and irinotecan. Among these chemotherapy agents, 5-FU is most commonly used for CRC [3]. Treatment with 5-FU has been shown to reduce tumor size by approximately 50% in patients with advanced CRC and prolong their median survival by 5 months [4]. However, since the late 1990s, studies have revealed that 5-FU treatment can lead to therapy resistance [5, 6] accompanied by hand-foot syndrome, cardiotoxicity [7], and gastrointestinal side effects [3]. Therefore, combination chemotherapy has become widely used in chemotherapeutic regimens [8]. Nevertheless, this therapeutic strategy might be partly beneficial; however, problems, such as new chemotherapeutic resistance or unacceptable side effects, can still occur. Therefore, explored of the resistance mechanism for 5-FU would benefit for searching the new safe and acceptable resistance-reversal medications.

In this case, natural products have great advantages because they have relatively fewer adverse effects. Herbal medicines applied to chemotherapy could improve chemotherapeutic efficacy and/or reduce induced toxicities in patients [9]. In addition, our previous studies have demonstrated that some herbal medicines suppressed cell growth, induced cell apoptosis, inhibited CRC angiogenesis, and overcame 5-FU resistance [10–12]. Therefore, herbal extracts might be feasible to increase chemotherapeutic efficacy and reduce the possibility of drug resistance and side effects.

*Prunella vulgaris* L. is a widely distributed perennial herb in the family Lamiaceae. In China, the spike of this herb, Spica Prunellae, is well known as a traditional Chinese medicine for its effects on heat clearing, dispersing swelling, and dissipating binds [13]. The herb has been reported to promote cancer cell apoptosis [14] and to suppress cell growth [15], vascular inflammation [16], tumor angiogenesis [17], and tumor volume [14, 18]; it has also been used in the treatment of cancers, such as breast cancer [19]. Recently, as a novel herbal medicine, LA16001, which is composed of *Prunella vulgaris* L., was found to prevent cisplatin-induced anorexia [20]. Extracts from other Lamiaceae plants, such as rosemary (*Rosmarinus officinalis*), have been shown to enhance the antitumor effect of 5-FU in 5-FU-resistant SW620-5FU-R cells [21]. Both *Rosmarinus officinalis* and *Prunella vulgaris* L. belong to the Labiatae family, and their marker compound is rosmarinic acid [22, 23], which has been shown to exhibit antioxidant [24], anti-inflammatory [25], and anticancer activities [26].

The aim of this study was to understand the resistance mechanism for 5-FU in depth and provide a clue for the searching of effective medications. Through bioinformatics analysis, DNA topoisomerase 2-alpha (TOP II alpha, TOP2*α*) was important in the 5-FU resistance. Moreover, we found it was involved in the 5-FU sensitivity enhancement of EESP. In the fact, Spica Prunellae was suggested as a potential reversal agent for treating 5-FU-resistant patients. In addition, searching for medications which could downregulate TOP2*α* expression would be a benefit clue for the therapy of 5-FU-resistant patients.

## 2. Materials and Methods

### 2.1. Identification of Key Genes Associated with 5-FU Sensitivity

We chose a public and freely available gene expression profile (GSE81005) from the US NCBI Gene Expression Omnibus database to screen differentially expressed genes between HCT-8/5-FU and HCT-8 cells treated following 5-FU treatment. We searched for differentially expressed genes firstly by comparing data between the treatment of 5-FU for 24/48 h in HCT-8/5-FU cells or HCT-8 cells and control groups, secondly comparing the differentially expressed genes between HCT-8/5-FU cells and HCT-8 cells, and thirdly obtaining the DEGs by searching the common DEGs between 24 h and 48 h treatment. DEGs were detected by the *R* (3.4.1) software. The adjusted *P* values were used to reduce the false-positive rate by using the Benjamini and Hochberg false discovery rate method by default. An adjusted *P* value of <0.05 was set as the cutoff criterion. Metascape was used to conduct pathway and process enrichment analysis of the DEGs using default settings [27]. STRING was employed to map the DEGs, which could detect the potential relationship among those DEGs. A maximum number of interactors = 0 and a confidence score ≥0.4 were set as the cutoff criteria. The Molecular Complex Detection (MCODE) app in Cytoscape was used further to screen modules in the PPI network with a cutoff = 2, *k*-core = 2, node score cutoff = 0.2, and max. depth = 100. Expression patterns of TOP2A in colon adenocarcinoma cancer and normal tissues were demonstrated by the available data from gene expression profiling interactive analysis (GEPIA) and R2 (https://hgserver1.amc.nl/cgi-bin/r2/main.cgi?&species=hs), which is a free publicly accessible web-based genomics analysis and visualization platform.

### 2.2. Transfection of siRNA or miR-494

siRNA and its siRNA control (Genepharma, Shanghai, China) or miR-494 mimics and miRNA negative control (Guangzhou RiboBio Co., LTD, Guangzhou, China) were synthesized. Cells were plated in 96 or 6 wells, and they were transfected with siRNA or miR-494 mimics at 50 nM at 30%–40% cell confluence following the instruction of Lipofectamine RNAiMAX (Thermo Fisher Scientific Inc., MA, USA). Then, cell viability was detected by performing a 3-(4,5-dimethylthiazol-2-yl)-2,5-diphenyltetrazolium bromide colorimetric assay (ELX800; BioTek, Winooski, USA) at 570 nm each day, or cells were collected for the western blot or gene expression validation in 48 h.

### 2.3. Western Blot

Proteins were extracted into RIPA buffer (CWBIO, Beijing, China), separated in 10% sodium dodecyl sulfate-polyacrylamide gel electrophoresis, and then transferred to 0.22 *μ*m NC membranes (Millipore, MA, USA). The membranes were blocked and probed with antibodies against TOP2*α*, *β*-actin, and the anti-rabbit IgG, HRP-linked antibody (Proteintech Group, Inc., Taiwan, China). The bands were detected with BeyoECL Plus (Beyotime Institute of Biotechnology, Shanghai, China) or Super ECL Star (US Everbright Inc., Suzhou, China). Image Lab Software (Bio-Rad, Hercules, CA, USA) was used to analyze the band intensity.

### 2.4. Gene Expression Validation

Total RNA was extracted with TRIzol (Takara, Dalian, China). Then, a nanodrop spectrophotometer (Thermo Fisher Scientific Inc., MA, USA) was used to assess the RNA quantity and quality. TOP2A primers for examination by real-time polymerase chain reaction (PCR) were designed from NCBI/Primer-BLAST, and GADPH primers were obtained from PrimerBank (https://pga.mgh.harvard.edu/primerbank/). The miRNA primers were obtained from (General Biosystems, NC, USA). Reverse transcriptase- (RT-) PCR was performed for miRNA examination by Mir-X™ miRNA First Strand Synthesis according to the SYBR® qRT-PCR User Manual (Takara, Dalian, China) and for mRNA detection by using a PrimeScript™ RT reagent Kit. Real-time PCR was outperformed by using a TB Green™ Premix Ex Taq™ II (Takara, Dalian, China) for miRNA and a SYBR™ Select Master Mix (Life Technologies, Shanghai, China). The 2-ΔΔCt method was used to analyze the expression levels.

### 2.5. EESP Stock Solution Preparation and Cell Culture

EESP stock solution was prepared as previously described [15]. Human carcinoma HCT-8 cells and HCT-8/5-FU cells (KeyGEN Biotech, Nanjing, China) were cultured at 37°C in a humidified incubator with 5% CO_2_ in RPMI medium 1640 (KeyGEN Biotech, Nanjing, China) supplemented with 10% FBS (Hyclone, Carlsbad, USA), 100 U/mL of penicillin, and 100 *μ*g/mL of streptomycin (Life Technologies, Shanghai, China). HCT-8/5-FU cells were cultured in the abovementioned medium with the addition of 15 *μ*g/mL of 5-FU (Shanghai Amino Acids Company, Shanghai, China).

### 2.6. Reversal Effect Assays

Two cell lines were seeded into 96-well plates at a density of 1 × 10^4^ cells/well. After 24 h, the cells were treated following various concentrations of EESP, 5-FU, or EESP plus 5-FU combination for 48 h. Then, the cell viabilities were evaluated. SPSS 16.0 software was used to calculate the cell survival rate and IC50 value. The resistance index (RI) of HCT-8/5-FU cells to EESP or 5-FU, reversal fold (RF), and relative reversal rate (RRR%) were calculated according to the following formulas:

  RI = IC50 of HCT-8/5-FU cells (EESP or 5-FU)/IC50 of HCT-8 cells (EESP or 5-FU)
  RF = IC50 of HCT-8/5-FU cells (5-FU)/IC50 of HCT-8/5-FU cells (EESP + 5-FU)
  RRR% = IC50 of HCT-8/5-FU cells (5-FU) − IC50 of HCT-8/5-FU cells (5-FU + EESP)/IC50 of HCT-8/5-FU cells (5-FU) − IC50 of HCT-8 cells (5-FU)

### 2.7. Colony Formation

The HCT-8 and HCT-8/5-FU cells were seeded into 6-well plates at a density of 4 × 105 cells/well. After transfection or treatment with 5-FU (3.2 mM) with/without EESP (0.25 mg/mL or 0.5 mg/mL) or EESP (0.25 mg/mL or 0.5 mg/mL) for 48 h, the cells were harvested and reseeded into fresh 12-well plates at a density of 500 cells/well. With 2 weeks of maintenance in RPMI medium 1640 supplemented with 10% FBS, penicillin, and streptomycin, the formed colonies were fixed with 4% polyoxymethylene and stained with 0.01% crystal violet.

### 2.8. Apoptosis Detection

After treatment with 5-FU (3.2 mM) and with/without EESP (0.25 mg/mL or 0.5 mg/mL) or EESP (0.25 mg/mL or 0.5 mg/mL) for 48 h in 6-well plates, apoptosis of cells was detected by using an AnnexinV-FITC Apoptosis Detection Kit (KeyGEN Biotech, Nanjing, China), as described in a previous study [15].

### 2.9. Statistical Analysis

Data are presented as the mean ± SD. The statistical significance of differences was assessed by Student's *t*-test or one-way ANOVA in SPSS 16.0 software. The level of statistical significance was set to ^*∗*^*P* < 0.05, ^*∗∗*^*P* < 0.01, and ^*∗∗∗*^*P* < 0.001.

## 3. Results

### 3.1. Identification of DEGs and Hub Genes

**Figure 1:**
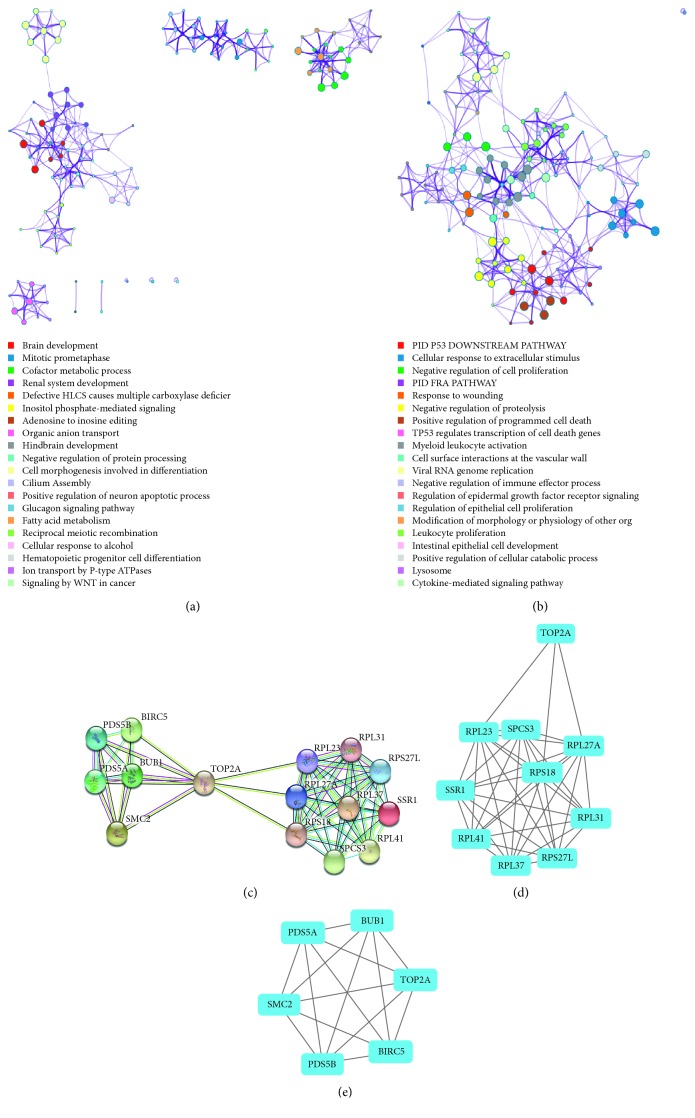
Identification of DEGs and hub genes. Enriched ontology clusters of (a) upregulated and (b) downregulated DEGs colored by cluster ID. (c) The PPI network of the top 15 hub genes. (d, e) Selection of the top 2 modules by using the MCODE plug-in to detect significant modules in this PPI network.

The potential molecular mechanisms were studied by searching for DEGs and hub genes. *R* (3.4.1) software was applied to detect DEGs in the US National Center for Biotechnology Information Gene Expression Omnibus GSE81005 dataset. Since the main difference between HCT-8/5-FU cells and HCT-8 cells was the 5-FU sensitivity, we analyzed hub genes during 5-FU intervention. We identified 1478 and 2316 DEGs in 5-FU treatment for 24 h and 48 h, respectively. We identified 582 DEGs in both the 24 h and 48 h 5-FU treatment groups. DEGs were functional and pathway enrichment analyzed using online tools in Metascape (http://metascape.org/), and the top 20 enrichment items were shown. As shown in Figures 1(a) and 1(b), the upregulated DEGs were enriched in brain development, mitotic prometaphase, cofactor metabolic process, etc., while the downregulated DEGs were enriched in PID P53 downstream pathway, cellular response to extracellular stimulus, negative regulation of cell proliferation, etc. To determine the essential genes involved in the 5-FU associated mechanism, the top 15 hub genes with a high degree of connectivity were screened (see [Supplementary-material supplementary-material-1]). Then, an associated PPI network was created on the basis of the information in the STRING database (Figure 1(c)). The top 2 modules were selected by MCODE to find the key modules in the PPI network. The results revealed that TOP2A was involved in both the modules (Figures 1(d) and 1(e)). This suggested that TOP2A might be a key gene for 5-FU resistance of colorectal cancer.

TOP2*α* was involved in the enhancement of 5-FU sensitivity to HCT-8/5-FU.

**Figure 2:**
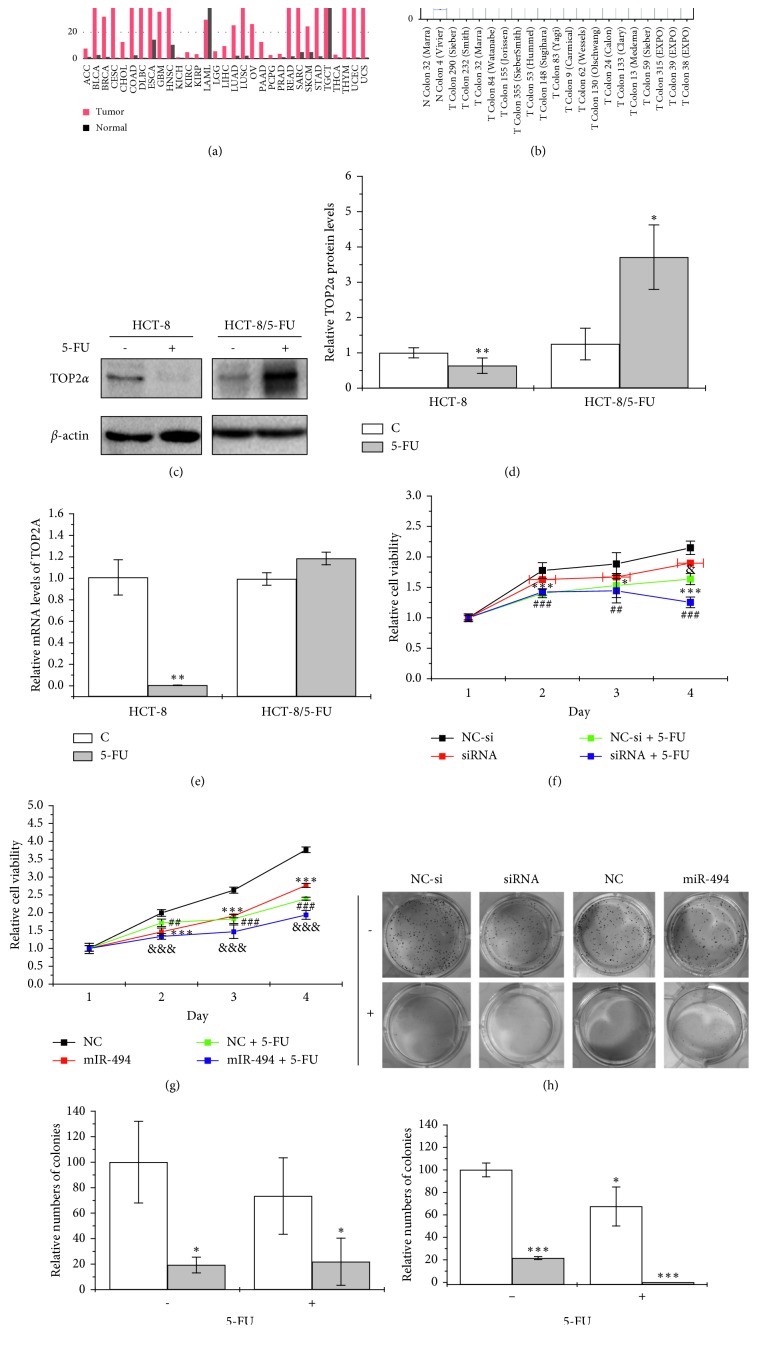
Overexpression of TOP2*α* mediates the 5-FU resistance. (a, b) Analysis of TOP2A expression levels in the GEPIA database and Mega-sampler in the TCGA database. (c) Detection of relative protein levels for HCT-8 and HCT-8/5-FU cells treated with or without 5-FU (3.2 mM) for 48 h by western blot and (d) quantification. (e) Real-time PCR evaluation of the mRNA levels of TOP2A. The data from western blot and real-time PCR are normalized to GADPH. Relative cell viability of HCT-8/5-FU cells transfected with (f) siRNA or (g) miR-494 and then treated with or without 5-FU (3.2 mM) for different time periods (1, 2, 3, and 4 days). (h) Cell colonies for HCT-8/5-FU cells transfected with siRNA or miR-494 and then treated with or without 5-FU (3.2 mM) for 48 h and (i, j) their relative numbers for cell colonies were analyzed. Compared with the negative control group, for siRNA or miR-494 transfection, ^*∗*^*P* < 0.05, ^*∗∗*^*P* < 0.01, and ^*∗∗∗*^*P* < 0.001; for negative control treatment with the 5-FU group (NC + 5-FU), ^#^*P* < 0.05, ^##^*P* < 0.01, and ^###^*P* < 0.001; for siRNA or miR-494 transfection and treatment with the 5-FU group (NC + 5-FU), ^&^*P* < 0.05, ^&&^*P* < 0.01, and ^&&&^*P* < 0.001.

As TOP2*α* was involved in both the modules, we speculate that it should be important in 5-FU resistance. Firstly, the expression levels of TOP2*α* were analyzed in a public database. Compared with normal tissues, the tumor samples appeared to have higher levels in the expression profiling of TOP2*α* (Figures 2(a) and 2(b)). These results suggested that overexpression of TOP2*α* might be a signal for tumor development. In view of this fact, we wondered if TOP2*α* overexpression might also be associated with 5-FU resistance. The expression levels of TOP2*α* between HCT-8 and HCT-8/5-FU cells exposed to 5-FU were compared. The results showed that TOP2*α* was significantly downregulated in HCT-8 cell lines but was upregulated in HCT-8/5-FU cell lines (Figures 2(c), 2(d), and 2(e)). To further confirm the role of TOP2*α* in 5-FU resistance, siRNA of TOP2*α* and miR-494 which was reported to target TOP2*α* [28] was used to knock down the expression of TOP2*α*. The efficiency of siRNA was shown in [Supplementary-material supplementary-material-1]. Compared to the negative control, siRNA or miR-494 transfection enhanced the 5-FU sensitive and cytotoxicity for HCT-8/5-FU cells (Figures 2(f) and 2(g)). Their transfection could also increase the cytotoxicity for HCT-8/5-FU cells, and miR-494 enhanced the 5-FU sensitivity for HCT-8/5-FU cells (Figures 2(h)–2(j)). But there was no significant difference in siRNA groups in the presence of 5-FU. These results indicated that TOP2A was involved in 5-FU resistance.

### 3.2. HCT-8/5-FU Cells Were Suitable for the Study of 5-FU Modulation with EESP

Given that the resistance index (RI) is a significant metric for evaluation of 5-FU-resistant cells to anticancer drugs, we examined the RI for EESP and 5-FU treatment. Compared with parental HCT-8 cells, HCT-8/5-FU cells showed a 257.4-fold increase in resistance to 5-FU and a 1.03-fold increase in resistance to EESP (Table 1). These results suggested that HCT-8/5-FU cells were resistant to 5-FU but not resistant to EESP; therefore, HCT-8/5-FU cells were suitable for the further study of 5-FU modulation with EESP.

### 3.3. Reversal Effect of EESP on 5-FU in HCT-8/5-FU Cells

For the evaluation of the reversal effect of EESP on 5-FU, two low concentrations of EESP (0.25 mg/mL and 0.5 mg/mL) with weakly cytotoxicity (inhibition rate < 20%) were chosen according to the evaluation of cell cytotoxicity with 5-FU in MTT assays. The results showed that EESP increased the sensitivity of HCT-8/5-FU cells to 5-FU by 3.88-fold (at 0.25 mg/mL) and 20.68-fold (at 0.5 mg/mL), respectively (Table 2). The relative reversal rates for these 2 concentrations of EESP were 74.56% and 95.53%, respectively. These results demonstrated that the combination of EESP with 5-FU increased the 5-FU sensitivity in HCT-8/5-FU drug-resistant cells although 0.25 mg/mL and 0.50 mg/mL of EESP combined separately with 5-FU each only partially reversed the effect of 5-FU.

### 3.4. EESP Enhanced 5-FU Cell Sensitivity in HCT-8/5-FU Cells via Cell Colony Formation Suppression and Cell Apoptosis Induction

**Figure 3:**
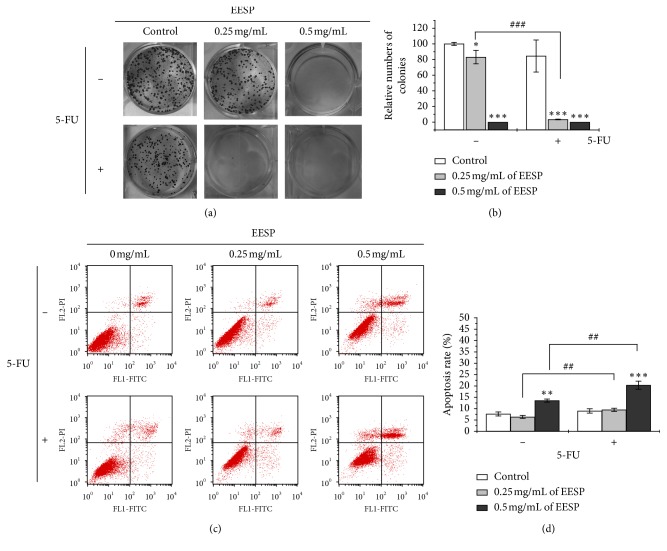
EESP combined with 5-FU suppressed the colony formation ability and induced apoptosis of HCT-8/5-FU cells. (a) After treatment with 5-FU (3.2 mM) with/without EESP (0.25 mg/mL or 0.5 mg/mL) or EESP (0.25 mg/mL or 0.5 mg/mL) for 48 h HCT-8/5-FU cells were fixed with methanol and stained with 0.01% crystal violet. (b) The numbers of colony formation were calculated from (a). (c) Treatment of HCT-8/5-FU cells with 5-FU (3.2 mM) with/without EESP (0.25 mg/mL or 0.5 mg/mL) or EESP (0.25 mg/mL or 0.5 mg/mL) for 48 h cell staining with annexin V/PI and analysis by using fluorescence-activated cell sorting. (d) Statistical analysis of the data from (c). Compared with the control group, ^*∗*^*P* < 0.05, ^*∗∗*^*P* < 0.01, and ^*∗∗∗*^*P* < 0.001; for 5-FU group, ^#^*P* < 0.05, ^##^*P* < 0.01, and ^###^*P* < 0.001.

To study the effects of EESP on the 5-FU sensitivity of colon carcinoma cells, 5-FU-resistant HCT-8/5-FU cells were treated at 0.25 mg/mL and 0.5 mg/mL of EESP separately with 5-FU at 3.2 mM or 0.25 mg/mL and 0.5 mg/mL of EESP alone for 48 h. There was no significant difference in the number of cell colonies between the 5-FU group and the control group. However, following treatment with 0.25 mg/mL and 0.5 mg/mL EESP combined with 5-FU, the number of cell colonies significantly decreased (Figures 3(a) and 3(c)). Furthermore, it was interesting that EESP in 0.25 mg/mL combined with 5-FU could enhance the suppression effect compared with 0.25 mg/mL of EESP alone groups. Similar results were also observed in the detection of cell apoptosis (Figures 3(b) and 3(d)). The combination of 5-FU with EESP in 0.5 mg/mL notably increased the apoptotic populations of HCT-8/5-FU cells relative to those in the 5-FU or EESP group. Results suggested that combination of EESP and 5-FU in low cytotoxicity concentration leads a better effect for enhancement of 5-FU sensitivity in HCT-8/5-FU cells. So in our following study, we chose to study its mechanism by EESP combined with 5-FU groups. These results together clearly implied that EESP enhanced 5-FU cell sensitivity in HCT-8/5-FU cells via suppression of cell colony formation and cell apoptosis induction.

### 3.5. ESSP Enhanced 5-FU Sensitivity in HCT-8/5-FU by Downregulating the Expression of TOP2*α*

**Figure 4:**
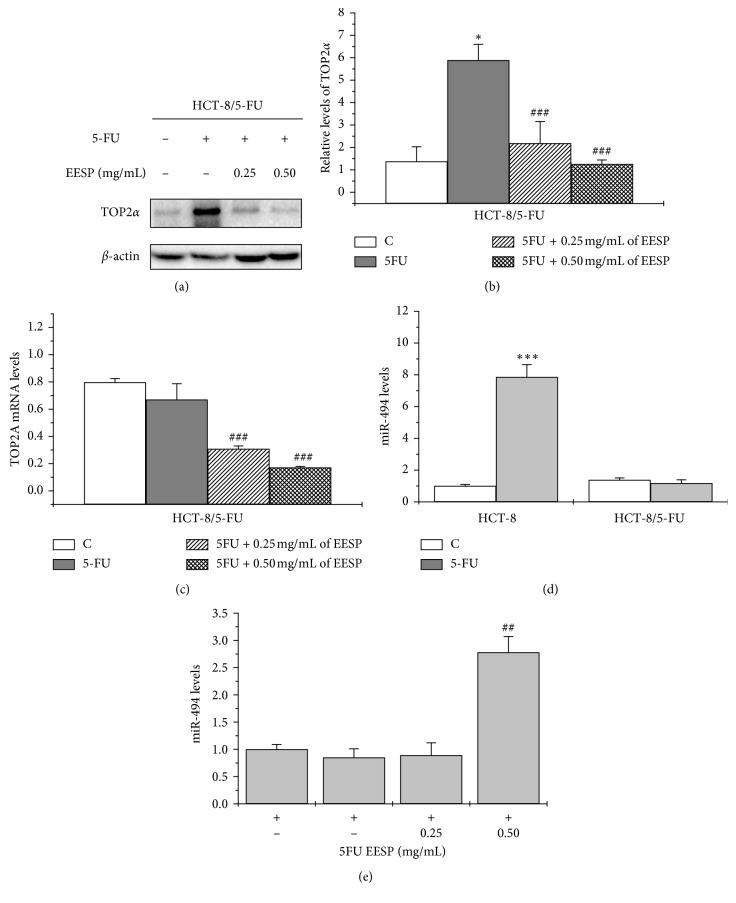
EESP enhanced the 5-FU sensitivity of HCT-8/5-FU cells through inhibition of TOP2*α*. (a) Detection of relative protein levels for HCT-8 and HCT-8/5-FU cells treated with 5-FU (3.2 mM), with or without EESP (0.25 mg/mL or 0.5 mg/mL), for 48 h by western blot and (b) quantification. (c) Real-time PCR evaluation of the mRNA levels of TOP2A. The data from western blot and real-time PCR were normalized to GADPH. (d) Detection of levels of miR-494 by real-time PCR in HCT-8 and HCT-8/5-FU cells treated with 3.2 mM of 5-FU for 48 h (e) Relative levels of miR-494 in HCT-8/5-FU cells treated with 5-FU (3.2 mM), with or without EESP (0.25 mg/mL or 0.5 mg/mL), for 48 h. Data were normalized to U6. Compared with control, ^*∗*^*P* < 0.05, ^*∗∗*^*P* < 0.01, and ^*∗∗∗*^*P* < 0.001; compared with the 5-FU (3.2 mM) treatment alone group, ^#^*P* < 0.05, ^##^*P* < 0.01, and ^###^*P* < 0.001.

Our results showed that EESP suppressed the expression of TOP2*α* protein significantly, which increased by 3.2 mM of 5-FU (Figures 4(a) and 4(b)). Similar results were shown in the transcription level analysis (Figure 4(c)). Because TOP2*α* was a downstream target for miR-494 [28], we decided to investigate its probable effects in the function of EESP. Levels of miR-494 were detected in HCT-8 and HCT-8/5-FU cells with or without 5-FU treatment. The expression level of miR-494 was increased following 3.2 mM 5-FU treatment in HCT-8 cells, but there was no difference in HCT-8/5-FU cells (Figure 4(d)). Furthermore, treatment with 0.5 mg/mL of EESP combined with 5-FU in HCT-8/5-FU cells significantly increased the levels of miR-494 (Figure 4(e)). In addition, combination with 5-FU seemed to enhance EESP effect for TOP2*α* and miR-494 ([Supplementary-material supplementary-material-1]), which was consistant with the results of cell colonies depression. Taken together, the results suggested that EESP suppressed TOP2*α* expression and promoted the levels of miR-494, which could partially reverse 5-FU resistance in HCT-8/5-FU cells.

## 4. Discussion

With the presence of 5-FU resistance, some chemical reversal molecules, such as bufalin [29], 2′,4′-dihydroxy-6′-methoxy-3′,5′-dimethylchalcone [30], oroxylin A [31], schizandrin A [32], and tyroservatide [33], were studied in 5-FU-resistant cell lines. However, these chemical agents might be restricted in clinical applications because of the single mechanism, poor selectivity, or unacceptable side effects.

As a chemotherapy agent, 5-FU is an analog of uracil [34] that inhibits thymidylate synthase and incorporates into DNA and RNA as the 5-FU metabolites [34]. 5-FU induces cell apoptosis through p53. Moreover, its sensitivity has been correlated with membrane-associated proteins, such as MDR3, MDR4, and MAT-8 [35, 36]. In addition, a recent study demonstrated that genes in drug metabolism-cytochrome P450 and pyrimidine metabolic pathways with promoter hypermethylation and concordant expression were silenced in HCT-8/5-FU cells [37]. Among fifteen hub genes in the PPI network, nine of them were related to protein synthesis and protein translocation. They consisted of seven genes associated with ribosome function: 5 ribosome proteins (RPL23, RPL27A, RPL37, RPL31, and RPL41) and 2 ribosome binding proteins (RPS18 and RPS27L). And the other two were translocation-associated proteins: signal peptidase complex subunit 3 and signal sequence receptor subunit 1. There were also three DNA replication-associated proteins (DNA topoisomerase TOP2A and sister chromatid cohesion proteins PDS5A and PDS5B). The rest of them were mitotic checkpoint serine/threonine kinase 1 (*BUB1* gene) which participated in mitosis, chromosome condensation protein of structural maintenance of chromosomes protein 2 (*SMC-2* gene), or baculoviral IAP repeat-containing 5 (*BIRC5 gene*). These genes indicated that DNA replication, protein synthesis, and protein translocation were active in 5-FU-resistant cells. Identifying the key protein might be a favorable way to reverse 5-FU resistance. In our study, TOP2*α* was selected since it was involved in both the modules. Besides, it was also reported as a character in new induction of 5-FU-resistant cell lines for its level was always altered in 5-FU-resistant cancers [38]. Furthermore, the other two hub genes, RPL37 and RPL23, were identified in the expression profiling of the cDNA-based microarray in response to 5-FU in a breast cancer cell [36] though without subsequent validation. So we considered that other genes were still valuable for further research. Additionally, because the resistance mechanism of 5-FU is complex as noted above and Chinese medicine with multiple compounds always targets many pathways, we suspected that EESP might have a role through other genes or pathways associated with 5-FU resistance besides regulation of TOP2*α*.

TOP2*α* is a DNA helicase encoded by the TOP2A gene, which is located in chromosome 17. TOP2*α* can instantaneously break and connect double-stranded DNA chains and regulate and alter the topological states of DNA during transcription and replication. Some type IIA topoisomerase inhibitors, including epipodophyllotoxins and anthracyclines, were selected for related diseases [39]. Mutation or overexpression of TOP2*α* was associated with chemotherapeutic resistance to some drugs, such as etoposide, irinotecan, and 5-FU [40–42]. In our study, we also confirmed the gene's pivotal role in 5-FU resistance by bioinformatics analysis. Besides TOP2*α*, topoisomerase-I, another protein from the topoisomerase family, has also been observed to be overexpressed in the tumor recurrences of patients with colorectal cancer who had received 5-FU-based adjuvant chemotherapy [42, 43]. We suggest that some other members in the topoisomerase family probably take part in the 5-FU-resistance mechanism or in the function of Spica Prunellae.

In our study, the protein level of TOP2*α* in HCT-8/5-FU cells after 5-FU exposure increased significantly, whereas its transcription level showed no difference from that in the control group (Figures 2(c)–2(e)). Since the levels of miR-494 was not change in the 5-FU exposure too (Figure 4(d)), it does not exclude the possibility that resistance to 5-FU for HCT-8/5-FU cells in this concentration of 5-FU was not in the activation of transcriptional levels but in the inhibition of protein degradation as a compensatory effect to protect cells. Of course, this hypothesis would be confirmed in the future by performing a cyclohexamide chase assay [44]. Moreover, in our study, we also observed that EESP could enhance 5-FU sensitivity of HCT-8/5-FU cells by cell apoptosis induction; considering that EESP contained multicompounds which helped it play multiroles, there must be some apoptosis pathways involved.

A recent study found that TOP2*α* is activated by Y-box binding protein-1 in transcriptional level, which is a multifunctional oncoprotein containing an evolutionarily conserved cold shock domain and dysregulates a wide range of genes involved in cell proliferation and survival, drug resistance, and chromatin destabilization by cancer [44]. So there is a possibility for EESP being suppressed by the expression of TOP2*α* via YBX1. Of course, this assumption needs to be confirmed by further investigation. Since our study suggested that TOP2*α* might be a marker for 5-FU resistant, it could be an index for selection of more effective treatments for 5-FU resistant patients. Additionally, we also recommend trying the medications like TOP2*α* inhibitors or other medicines like Spica Prunellae (with less side effects and charges) in the clinical therapy might achieve some new hope to change the bottleneck period for 5-FU resistance.

MicroRNAs (miRNAs) are a group of small noncoding RNA molecules. They are crucial in every period of life or cancer development. Previous studies have demonstrated that TOP2*α* was one of the targets for miR-494 [28]. miR-494 has a global regulatory role in the cell cycle process by binding to the open reading frame and downregulating TOP2*α* and PTTG1 [28]. Fortunately, we noticed that EESP enhanced 5-FU sensitivity by downregulating TOP2*α* and miR-494. We suggest that EESP probably suppressed expression of TOP2A via upregulation of miR-494. This mechanism will be confirmed by miRNA mimics and inhibitors in a planned future study. Furthermore, because more than two of these miRNAs are involved in the regulation of TOP2*α* or drug-resistance mechanisms, they would be valuable to study in the future.

## 5. Conclusions

Chemotherapy is a common phenomenon during the treatment of colorectal cancer. In our study, we aimed to find some key genes involved in chemotherapy resistance of colorectal cancer. An important gene, *TOP2A*, was obtained for 5-FU resistance of colorectal cancer by bioinformatics analysis. Our study showed that TOP2*α* is a potentially important protein in the 5-FU-resistance mechanism and Spica Prunellae partly reversed 5-FU resistance by downregulating TOP2*α* and upregulating levels of miR-494. Furthermore, we believe that Spica Prunellae has more advantages in clinical therapy for 5-FU-resistant patients because it is safer and has fewer adverse effects than chemical agents have.

## Figures and Tables

**Table 1:** Inhibitory effects of EESP and 5-FU on HCT-8 and HCT-8/5-FU cells for 48 h (*n* = 3).

Cell line	^a^IC_50_	
	EESP (mg/mL)	5-FU (mM)
HCT-8	0.77 ± 0.07	0.60 ± 0.13
HCT-8/5-FU	0.75 ± 0.09	154.46 ± 14.07
RI	1.03	257.40

^a^IC_50_ represents semi-inhibitory concentration of modulators.

**Table 2:** Potency of EESP in enhancing cytotoxicity of 5-FU in HCT-8/5-FU cells (*n* = 3).

Anticancer drugs	IC_50_	RF^a^	RRR%^b^
0.25 mg/mL EESP + 5-FU	49.46 ± 2.14	3.88	74.56
0.50 mg/mL EESP + 5-FU	7.47 ± 1.91	20.68	95.53

^a^RF and ^b^RRR% represent reversal effect of modulators. The greater the RF magnitude, the more significant the effect. When RRR% ≥100%, the modulator totally reversed the effect of 5-FU; when RRR% <100%, the modulator only partially reversed the effect of 5-FU.

## Data Availability

The gene expression profile data supporting this ontology clusters enrichment and PPI work are from previously reported studies and datasets, which have been cited. The processed data are available at the US NCBI Gene Expression Omnibus database. The expression patterns data supporting expression patterns of TOP2A in colon adenocarcinoma cancer and normal tissues are from previously reported studies and datasets, which have been cited. The processed data are available at gene expression profiling interactive analysis (GEPIA) and R2 (https://hgserver1.amc.nl/cgi392bin/r2/main.cgi?&species=hs).

## Supplementary Materials

Supplementary Materials Table S1: top 15 hub genes with higher degree of connectivity. Figure S1: transfection of siRNA and miR-494 downregulated levels of TOP2*α* in HCT-8/5-FU cells. (A) miR-494 levels were detected by real time PCR after transfection of its mimics for 48 h. (B) Analysis of TOP2A mRNA levels. (C) TOP2*α* protein levels in HCT-8/5-FU cells after transfection of siRNA and miR-494 mimics for 48 h. (D) Quantification of (C). Compared with the negative control group, ^*∗*^*P* < 0.05, ^*∗∗*^*P* < 0.01, and ^*∗∗∗*^*P* < 0.001.
