# Supplementary material for: Spica Prunellae Extract Enhances Fluorouracil Sensitivity of 5-Fluorouracil-Resistant Human Colon Carcinoma HCT-8/5-FU Cells via TOP2α and miR-494
Source: Biomed Res Int. 2019 Sep 30;2019:5953619. doi: 10.1155/2019/5953619 (PMC6791265; doi:10.1155/2019/5953619)
Supplement: Supplementary Materials — Table S1: top 15 hub genes with higher degree of connectivity. Figure S1: transfection of siRNA and miR-494 downregulated levels of TOP2α in HCT-8/5-FU cells. (A) miR-494 levels were detected by real time PCR after transfection of its mimics for 48 h. (B) Analysis of TOP2A mRNA levels. (C) TOP2α protein levels in HCT-8/5-FU cells after transfection of siRNA and miR-494 mimics for 48 h. (D) Quantification of (C). Compared with the negative control group, ∗P < 0.05, ∗∗P < 0.01, and ∗∗∗P < 0.001. [file 5953619.f1.doc]

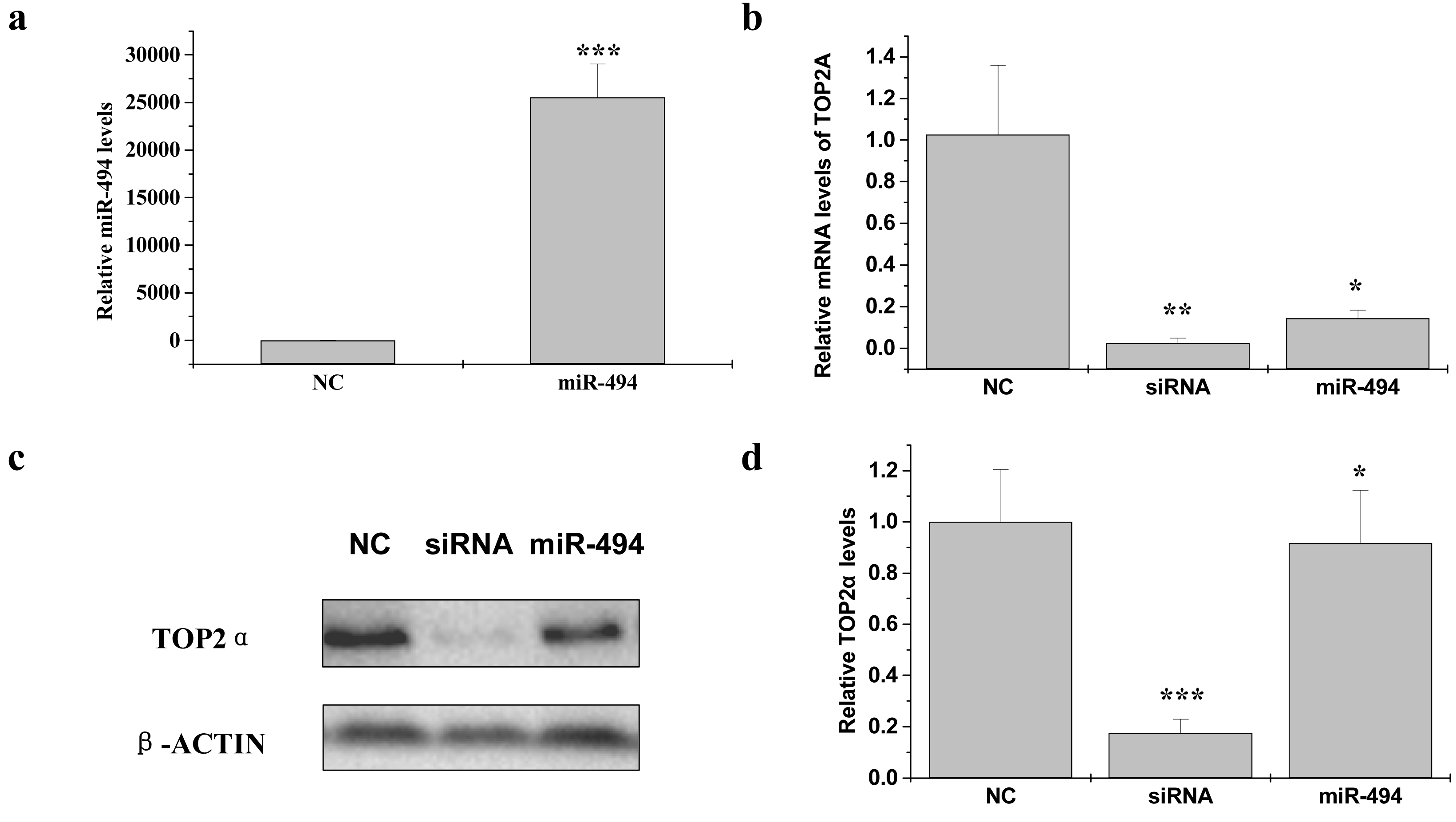


Figure S1 Transfection of siRNA and miR-494 downregulated levels of TOP2α in HCT-8/5-FU cells. (A) miR-494 levels were detected by real time PCR after transfection of its mimics for 48 h. (B) Analysis of TOP2A mRNA levels and (C) TOP2α protein levels in HCT-8/5-FU cells after transfection of siRNA and miR-494 mimics for 48 h. (D) quantification of (C). Compared with negative control group, **P* < 0.05, ***P* < 0.01, and ****P* < 0.001.

Table S1 Top 15 hub genes with higher degree of connectivity

| Rank | Name | Score |
| --- | --- | --- |
| 1 | RPL23 | 50454 |
| 2 | RPL27A | 50448 |
| 3 | RPS18 | 50432 |
| 4 | RPS27L | 50408 |
| 5 | RPL37 | 50406 |
| 6 | RPL31 | 50400 |
| 6 | RPL41 | 50400 |
| 8 | SPCS3 | 40321 |
| 9 | SSR1 | 40320 |
| 10 | BIRC5 | 8231 |
| 11 | TOP2A | 7489 |
| 12 | BUB1 | 6242 |
| 13 | SMC2 | 6026 |
| 14 | PDS5A | 5795 |
| 15 | PDS5B | 5785 |

Table S1 Top 15 hub genes with higher degree of connectivity

| Rank | Name | Score |
| --- | --- | --- |
| 1 | RPL23 | 50454 |
| 2 | RPL27A | 50448 |
| 3 | RPS18 | 50432 |
| 4 | RPS27L | 50408 |
| 5 | RPL37 | 50406 |
| 6 | RPL31 | 50400 |
| 6 | RPL41 | 50400 |
| 8 | SPCS3 | 40321 |
| 9 | SSR1 | 40320 |
| 10 | BIRC5 | 8231 |
| 11 | TOP2A | 7489 |
| 12 | BUB1 | 6242 |
| 13 | SMC2 | 6026 |
| 14 | PDS5A | 5795 |
| 15 | PDS5B | 5785 |
